# Needle: a fast and space-efficient prefilter for estimating the quantification of very large collections of expression experiments

**DOI:** 10.1093/bioinformatics/btac492

**Published:** 2022-07-08

**Authors:** Mitra Darvish, Enrico Seiler, Svenja Mehringer, René Rahn, Knut Reinert

**Affiliations:** Efficient Algorithms for Omics Data, Max Planck Institute for Molecular Genetics, Berlin, Germany; Efficient Algorithms for Omics Data, Max Planck Institute for Molecular Genetics, Berlin, Germany; Algorithmic Bioinformatics, Institute for Bioinformatics, FU Berlin, 14195 Berlin, Germany; Algorithmic Bioinformatics, Institute for Bioinformatics, FU Berlin, 14195 Berlin, Germany; Efficient Algorithms for Omics Data, Max Planck Institute for Molecular Genetics, Berlin, Germany; Efficient Algorithms for Omics Data, Max Planck Institute for Molecular Genetics, Berlin, Germany; Algorithmic Bioinformatics, Institute for Bioinformatics, FU Berlin, 14195 Berlin, Germany

## Abstract

**Motivation:**

The ever-growing size of sequencing data is a major bottleneck in bioinformatics as the advances of hardware development cannot keep up with the data growth. Therefore, an enormous amount of data is collected but rarely ever reused, because it is nearly impossible to find meaningful experiments in the stream of raw data.

**Results:**

As a solution, we propose Needle, a fast and space-efficient index which can be built for thousands of experiments in <2 h and can estimate the quantification of a transcript in these experiments in seconds, thereby outperforming its competitors. The basic idea of the Needle index is to create multiple interleaved Bloom filters that each store a set of representative *k*-mers depending on their multiplicity in the raw data. This is then used to quantify the query.

**Availability and implementation:**

https://github.com/seqan/needle.

**Supplementary information:**

Supplementary data are available at *Bioinformatics* online.

## 1 Introduction

The advances of whole genome sequencing technologies have led to an exponential increase in sequencing data, and the amount of collected data already exceeds several databases (https://www.ncbi.nlm.nih.gov/sra/docs/sragrowth/, last time accessed: 24 February 2021). Especially in the domain of RNA-sequencing, researchers want to quantify transcripts in their analysis (Seiler *et al.*, 2021). The analysis of existing sequencing experiments could be helpful for a variety of reasons, including allowing researchers to (i) find leads to promising new research topics or (ii) perform a sanity check of their findings.

For instance, if sequencing experiments were easily searchable by their quantification, differential gene expression analysis among different conditions, genomes, tissue, etc. could be performed on all existing sequencing data and could result in several possible genes involved in the researched condition, which then can be the starting point of a more in-depth research project. The same approach could be used to determine whether a gene set known for a specific biological process is also involved in other processes. Alternatively, such a large-scale quantification can be useful to verify findings based on a small-scale analyses.

Therefore, quantifying massive collections of sequencing experiments can open the door to a better and wider understanding of transcripts and their biological meaning by making databases searchable, even if this means to allocate additional space on the respective servers.

However, extracting relevant information from a large amount of raw data is currently not possible in reasonable time and space. A standard procedure to narrow down the experiments to those relevant for a study is to scan the associated metadata. But this procedure only works effectively if the metadata is complete and consistent, which is often not the case. Furthermore, the metadata is not systematically updated to newer findings, so that any recently discovered transcripts of interests cannot be found. Moreover, metadata searches usually do not contain any quantification information. Therefore, space-efficient and fast search algorithms that can quickly (re-)analyze and filter the raw sequencing data are needed to identify data of interest.

In the last few years, several tools for indexing a large amount of sequencing data were developed. These tools are based on the analysis of the underlying set of *k*-mers (Bingmann *et al.*, 2019; Harris and Medvedev, 2020; Kitaya and Shibuya, 2021; Lemane *et al.*, 2021; Marchet *et al.*, 2020; Pandey *et al.*, 2018; Seiler *et al.*, 2021; Solomon and Kingsford, 2016, 2018; Sun *et al.*, 2018; Yu *et al.*, 2018). The main idea is to store the *k*-mers of a representative subset [e.g. minimizers (Marçais *et al.*, 2017)] of the sequencing reads in a space-efficient data structures such as counting quotient filters (Pandey *et al.*, 2018), Bloom filters (e.g. Solomon and Kingsford, 2016) or interleaved Bloom filters (IBFs) (Seiler *et al.*, 2021). Despite being groundbreaking, all of these tools can only answer simple membership queries, with no ability to quantify found transcripts.

REINDEER (Marchet *et al.*, 2020) was the first tool to not only store representative *k*-mers for all given experiments but also how often they occur. These so-called count values can either be exact or approximate. Although a major breakthrough, REINDEER does not offer an actual estimation of the quantification for a transcript, but leaves it to the user to interpret the stored count values.

Recently, the tool Gazelle (Zhang *et al.*, 2021) was published. Similar to REINDEER, Gazelle stores *k*-mers and their counts, but unlike REINDEER, they always perform a log-transform of the count values to save space, claiming that storing the raw count values is not necessary as significant differences in gene expression data often follow a double log fold change (Zhang *et al.*, 2021). Furthermore, Gazelle offers an estimation of the quantification for a transcript by taking the interquartile mean of the stored count values.

Traditionally, quantification involves a computationally expensive alignment step, where the reads of one RNA-sequencing (RNA-seq) experiment are aligned to a transcriptome to measure the expression, e.g. STAR (Dobin *et al.*, 2013). Lately, alternative approaches have been proposed, where this alignment step was replaced by faster methods such as analyzing the *k*-mers of a transcript [e.g. Sailfish (Patro *et al.*, 2014)] or using pseudoalignments [e.g. the tools kallisto (Bray *et al.*, 2016) and Salmon (Patro *et al.*, 2017)]. All of these approaches use an expectation–maximization algorithm to handle ambiguity of reads or *k*-mers and a transcriptome to determine relevant *k*-mers beforehand. Although these developments have been a considerable improvement compared to exact alignments, analyzing a dataset of thousands of experiments is still too costly.

In this article, we introduce Needle, a tool for semi-quantitative analysis of large collections of expression experiments. Needle is based on two ideas. First, it uses the IBF (Dadi *et al.*, 2018; Seiler *et al.*, 2021) with minimizers instead of contiguously overlapping *k*-mers to efficiently index and query these experiments. Second, rather than storing the exact raw count value of every minimizer, Needle splits the count values of one experiment into meaningful buckets and stores each bucket as one IBF.

We show how this discretization can efficiently and accurately approximate the expression of given transcripts for all given files at once. Due to the efficiency of the IBF, Needle can build the index 3−54 times faster than REINDEER and the count values can be obtained 16−100 times faster while only using 3−39% of the space required by REINDEER, where the speed advantage depends on the chosen minimizer window size. A direct comparison to Gazelle was not possible as the tool is not publicly available yet, but based on their provided analysis of Gazelle (Zhang *et al.*, 2021), we are quite confident that Needle also outperforms Gazelle.

## 2 Materials and methods

### 2.1 Minimizers

Alignment-free methods rely on a simpler, but still representative method to capture similarities of sequences. Most common is the usage of all *k*-mers, but similar *k*-mers often contain the same information. Therefore, it is not necessary to store all of them. For this reason, *(w, k)-minimizers* (Roberts *et al.*, 2004; Schleimer *et al.*, 2003) are used in Needle.

**Fig. 1.**
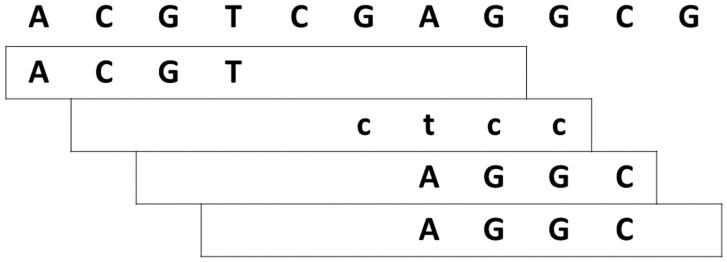
Example of (8, 4)-minimizers. The shown 4-mers are the smallest 4-mers in their respective window of size 8. The second minimizer originates from the reverse complement strand and is denoted by lower letters and has to be read from right to left. The last two windows share the same minimizer, as it is often the case for subsequent windows. They are shown twice, but are only stored once

A minimizer is the smallest *k*-mer of all *k*-mers (including those on the reverse complement strand) inside a window of length *w* (see Fig. 1 for an example). Often, the minimizer of one window will remain the minimizer (the smallest *k*-mer) when shifting the window by one. In that case, the minimizer is stored only once, which leads to a reduction of memory cost for minimizers compared to simple *k*-mers.

Notably, we also use (w,k)−minimizers where the window size *w* is equal to *k*. In this case, there is still the choice between the *k*-mers of both strands. Hence, (k,k)−minimizers represent canonical *k*-mers.

In Figure 1, the smallest *k*-mer is defined by the lexicographical ordering, but it has been shown that prior randomization is beneficial as it prevents a skewed distribution (Marçais *et al.*, 2017).

For the properties and the size of the data we will handle, *k* will usually be in the range of 16−32 because in this range the chance of random hits is rather low.

### 2.2 The Interleaved Bloom Filter

An IBF (Dadi *et al.*, 2018; Seiler *et al.*, 2021) with *b* bins consists of *b* Bloom filters (Bloom, 1970) which then are interleaved.

A Bloom filter is a probabilistic data structure based on a bitvector and *h* hash functions. To insert a value (minimizers), the bits pointed to by the *h* hash functions are set to one. The minimizers are treated as integers and the hash functions are based on fastrange (Lemire, 2019) and different large irrational numbers as seeds to ensure uniform hashing. A value is considered to be present in a Bloom filter if all of its hash positions are set.

Because different input values can have the same hash values for some hash functions, a query answer of a Bloom Filter might be a false positive. The bigger the Bloom Filter, the smaller the false positive rate. The probability of a false positive given a Bloom filter of size *n* bits and *m* inserted elements is approximately

(1) $$p_{f}={\left(1-{\left(1-\frac{1}{n}\right)}^{h\cdot m}\right)}^{h}.$$

**Fig. 2.**
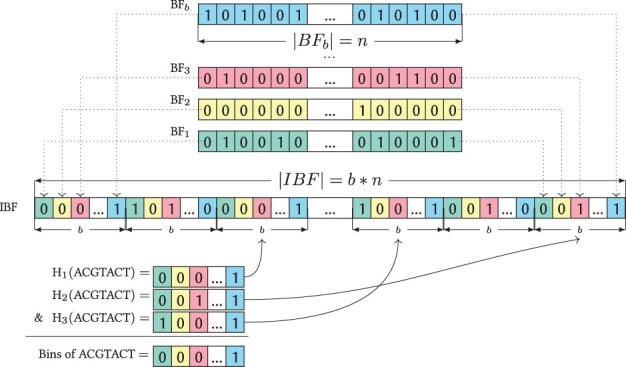
Example of an IBF. At the top, individual Bloom filters of length *n* are shown, these were interleaved to create an IBF of size *b *×* n* using three hash functions. When querying one minimizer, here ACGTACT, the hash functions return three positions in the IBF, so that three sub-bitvectors can be retrieved. These sub-bitvectors are combined with a bitwise & to a final resulting bitvector, where a 1 indicates that the minimizer is found in that experiment. Here, ACGTACT can be found in the last (blue) experiment. The figure is taken from Dadi *et al.* (2018)  (A color version of this figure appears in the online version of this article.)

While one Bloom filter can only store information about one experiment, the IBF can contain *b* experiments by interleaving *b* Bloom filters. Essentially, an IBF replaces each bit in the Bloom filter with a bitvector of size *b*, where the *i*-th bit contains information about the *i*-th experiment. Hence, an IBF consists of b·n bits. Inserting the information on a single experiment *i* works similar to the insertion into a Bloom filter: the hash functions return the start positions to a *b*-sized bitvector and the *i*-th bit of this subvector is set. Therefore, retrieving all experiments containing a certain value is easy: All *b*-sized bitvectors that the *h* hash functions point to are combined into a final bitvector by using a logical AND operation. A set bit in such a final result vector indicates that the experiment contains the searched value (see Fig. 2 for an example).

These resulting bitvectors can be used to create count vectors by simply accumulation.

Note that an IBF is similar to a Bloom filter at its core, as both are bitvectors and therefore are easy to compress using sparse bitvectors, at the cost of increasing the running time of a query.

### 2.3 Quantification

**Fig. 3.**
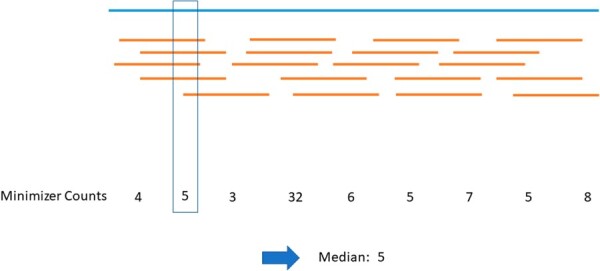
Example how the expression value is derived. The blue line stands for a transcript, while the yellow lines symbolize reads covering this transcript. As we do not use an alignment step, this information is not available to Needle. The only available information is the number of minimizer counts in the file containing the reads. For example, the 5 reads intersecting the box could each have a minimizer in the box which would result in a count of 5. If a minimizer is unique to a transcript, the minimizer counts represent the amount of reads covering the transcript. If the minimizer is not unique to a transcript, but also appears in a different transcript, the minimizer count might be higher (e.g. 32 in the example). Taking the median of these minimizer counts disregards the outliers and results in a reliable expression value (A color version of this figure appears in the online version of this article.)

The main goal when quantifying a query usually is to be as accurate as possible while still being fast and space-efficient. For a prefilter, this goal is reversed because a more in-depth analysis with more accurate methods can follow once relevant experiments have been extracted. Therefore, speed and space-efficiency are the main motivators for Needle. On that account, we define the expression value of a transcript as the median of its minimizer counts. We chose the median instead of the mean to disregard outliers (see Fig. 3 for an example).

This approach is potentially less accurate than a (pseudo-) alignment approach, as no alignment is performed and all minimizers, no matter what their most likely origin is, are considered and all information of their order is lost. Hence, isoforms or paralogous genes might be harder to distinguish from each other. However, if parts of a transcript are not covered at all or covered extensively, it will not impact the expression value because the median is stable against outliers.

We will show that this approach is still accurate enough to serve as an efficient prefilter and its results are close to the actual expression values in most cases.

### 2.4 The q-quantitative filter

**Fig. 4.**
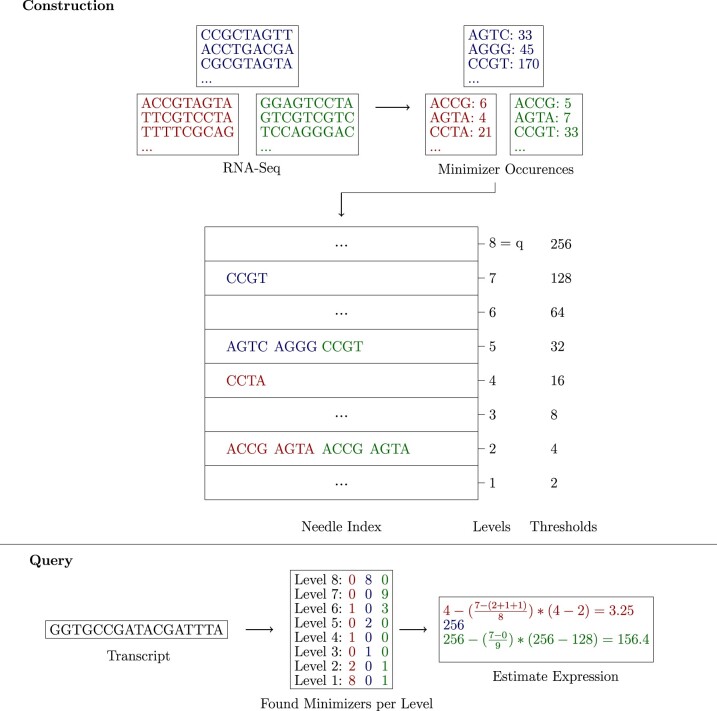
Example of Needle’s workflow for (4, 4)-minimizers. The q-quantitative filter is constructed by determining all minimizers and their occurrences. A minimizer *z* with count *c* is added to level *i* if $t_{i}<c\leq t_{i+1}$. For example, minimizer ‘ACCG’ occurs six times in the red file and is stored in the second level, because $t_{2}=4<=6<t_{3}=8$. Note that the dots in the otherwise empty levels represent minimizers not shown in the small excerpt of the RNA Seq files. The query transcript consists of 17 nucleotides and has 14 minimizers. If half of that (7) are found for any color, the transcript’s expression is estimated. For the red example, this happens on the first level. For the blue example, on the eighth level (because this is the last level, the expression is set to the threshold of that level) and, for the green example, on the seventh level (A color version of this figure appears in the online version of this article.)

To store count values efficiently, we use *q* IBFs to discretize the count value distribution. An IBF *i* contains only minimizers with count values greater or equal than *t_i_* and smaller than $t_{i+1}$, where *t_i_* and $t_{i+1}$ are called thresholds. There are *q* levels in total. For the last level, all minimizers greater or equal to its threshold *t_q_* are stored (see Fig. 4 for an example).

Determining good thresholds for RNA-seq experiments is challenging because the minimizer content differs for each experiment. Furthermore, the minimizer count distribution does not contain multiple local minima, meaning that there is no obvious approach to partition the distribution.

Needle provides two options for defining the thresholds. The first option consists of *user-defined thresholds*. The second option is to use *automatic thresholds* for each individual experiment. Instead of having the same threshold for each experiment on one level, each experiment has its thresholds which are determined by recursively computing the median of the number of minimizer occurrences to determine *t_i_*, stopping once *q −* 1 thresholds are determined. Hence, the minimizer content at every level is half of the minimizer content at the previous level (except for the last level, which contains all minimizers with counts $>t_{q}$).

As this would lead to disregarding every minimizer smaller than the first threshold, the first threshold is defined by a cutoff. The cutoff is either user-defined or automatically determined by the file size, similar to the cutoffs used in other tools (Pandey *et al.*, 2018; Solomon and Kingsford, 2016) ($t_{1}=$ cutoff).

The advantage of the *user-defined thresholds* is that the thresholds do not need to be computed and take less space because there are only *q* thresholds for all experiments. However, the *automatic thresholds* are based on the actual minimizer count distribution of the experiments.

#### 2.4.1 Constructing the q-quantitative filter

The size of an IBF is crucial for the false positive rate and should therefore be picked carefully. Needle expects the user to define a desired false positive rate and sets the size of one IBF according to the average number of minimizers that needs to be stored per experiment. If the *user-defined thresholds* are used, it is necessary to check the distribution of minimizers over the given levels for each experiment. If the *automatic thresholds* are applied, the expected number of minimizers can be estimated from the total number of minimizers in a file by recursively dividing that number by two for every level.

As experiments can have a highly diverse number of minimizers, especially if the experiments have different coverages, the false positive rate will not hold for every experiment, only for those with a number of minimizers close to the average. To take the false positive rate into account while querying, the actual false positive rate for every experiment is stored in an extra output file.

Once the sizes of all IBF s are known, they are created simultaneously and kept in main memory, while going over every experiment and inserting the minimizers in the correct IBF according to their counts.

#### 2.4.2 Querying the q-quantitative filter

To answer the query of how much a given transcript is expressed in each of the target experiments, we count the number of (*w*, *k*)-minimizers of the transcript at each level using the respective IBFs. Given that the expression of a transcript is defined as the median of its occurring minimizer count values, the expression value can be obtained from the count values’ median. However, the actual count values are not stored, and the median must be approximated. In the following, we describe how this can be achieved by a linear scan over all *q* IBFs of the q-quantative filter.

Let *T* be some transcript and T′ be the set of all (*w*, *k*)-minimizers therein, with |T′|=m. Furthermore, let $C_{i}(T\prime )$ be the number of minimizers ∈T′ found in the IBF at level *i*. Using this, we can approximate the median (= expression value) *μ_e_* for each experiment *e* in the q-quantitive IBF by a linear interpolation of two adjacent levels, indicated by the indices *x_e_* and $y_{e}=x_{e}+1$. The index of *x_e_* is chosen such that

(2) $$\begin{array}{c} a_{e}=C_{x_{e}}(T\prime )\\ b_{e}=\sum_{i=y_{e}}^{q}C_{i}(T\prime )\\ a_{e}+b_{e}\geq \frac{m}{2}\wedge b_{e}<\frac{m}{2}. \end{array}$$

That is, the index of the level at which the sum of all counts in this level (*a_e_*) and all levels above (*b_e_*) is equal to or greater than half of the number of (*w*, *k*)-minimizers present in *T* ($\frac{m}{2}$) and, in addition, the count of all levels above (*b_e_*) is less than this number. From this, the median *μ_e_* can be approximated by

(3) $$\mu_{e}=y_{e}-(y_{e}-x_{e})*\left(\frac{m-b_{e}}{a_{e}}\right).$$

To calculate *b_e_*, the number of found minimizers at levels higher than *y_e_* has to be considered, otherwise the threshold of $\frac{m}{2}$ might never be met. This is problematic as every IBF has its own false positive rate. Therefore, summing up the results of multiple IBFs would lead to a higher false positive rate and makes a correction necessary. The number of observed minimizers at one level (*n_o_*) is the result of true positives (*n_tp_*) and false positives, and it can be assumed that

(4) $$n_{o}=n_{tp}+(n-n_{tp})*\mathrm{fpr}$$

holds for *n* being the number of minimizers in a transcript and *fpr* the false positive rate of the underlying IBF for one specific experiment. By transforming this formula, the number of true positives can be estimated by:

(5) $$n_{tp}=\frac{n_{o}-n*\mathrm{fpr}}{1-\mathrm{fpr}}$$

In summary, each of the *q* IBFs created is searched once to find the two levels *x* and *y*. As a result of this, the search time is *O*(*q*). Although this seems like a drawback, in reality, a small number of levels q≤15 is sufficient and our current search speed outperforms REINDEER by a factor of 27−1205 (see Table 2).

**Table 2.** Comparison of Needle and REINDEER for *k *=* *21 on the large real dataset

		Build			Query			
		Time	RAM	Index size	1	100	1000	RAM
1 Thread	REINDEER	—	—	—	813	897	1710	80.7
	REINDEER log	432	39.4	27.9	>559	>559	>559	67.3
	Needle (21, 21)	112 (118)	121.1	62.2 (20.1)	48 (20)	40 (33)	141 (208)	30.4 (9.7)
	Needle (25, 21)	33 (37)	38.4	19.7 (6.9)	11 (8)	13 (12)	48 (68)	9.6 (2.9)
	Needle (41, 21)	9 (8)	9.4	4.8 (1.7)	2 (3)	4 (3)	15 (20)	2.4 (0.7)
4 Threads	REINDEER	251	44.1	50.5	>746	>746	>746	80.7
	REINDEER log	218	39.4	27.9	>559	>559	>559	67.3
	Needle (21, 21)	34 (33)	121.1	62.2 (20.1)	23 (12)	19 (17)	45 (62)	30.4 (9.7)
	Needle (25, 21)	9 (11)	38.4	19.7 (6.9)	9 (5)	10 (7)	19 (19)	9.6 (2.9)
	Needle (41, 21)	3 (3)	9.4	4.8 (1.7)	2 (1)	3 (2)	5 (6)	2.4 (0.7)

*Note*: For the query comparison, the run times for 1/100/1000 sequence(s) were measured. The RAM usage was the same for all query comparisons. The REINDEER build with one thread resulted in a zlib error, the REINDEER log query resulted in a malloc error and the REINDEER query with 4 threads in a segfault, in these cases the time for loading the index is reported. Needle (w,k) represents Needle based on (w,k)-minimizers. Build-Time is in minutes, Query-Times in seconds, RAM, and Index size in GB. The values in parentheses are the results when using compressed IBFs.

The described search could either start with IBF_1_ and then search IBFs with increasing levels, or start with the IBF_*q*_ and then search downwards. IBF_1_ depends on either the first user-defined threshold or the cutoff value, which are usually not set to 0 to exclude minimizers resulting from erroneous reads. Therefore, Needle starts with IBF_*q*_ because then it is not necessary to account for this dependency.

#### 2.4.3 Normalization

Normalization is an important step in any further downstream analysis, but not every normalization method is affordable for a large dataset. For instance, a normalization method considering all available data for the normalization [like DESeq2 (Love *et al.*, 2014)] becomes computationally expensive when using hundreds of experiments. Therefore, Needle provides a normalization which is based on the content of one experiment [like FPKM, TPM values (Zhao *et al.*, 2021)]. The estimated expression value is normalized by dividing it by the value of the threshold of the second level *t*_2_ of that experiment. Because the levels depend on every experiment and their minimizer content, higher sequencing coverages lead to a greater divisor and thereby to a more accurate comparison between the experiments. (Level 1 and its thresholds *t*_1_ are ignored because they are based on the cutoff.) Notably, unlike FPKM or TPM, the presented approach does not need a correction for the gene length because the determination of the median is independent of the number of minimizers. Furthermore, this normalization method only works for individual thresholds and not user-defined thresholds because it is based on different thresholds per experiment.

### 2.5 Accuracy evaluation

To evaluate the accuracy of Needle’s approach, simulated and real experiments were used to measure the quantification step by considering different expressions of transcripts in one sample and across multiple samples.

All methods were tested with Needle(v1.0.1) and its direct competitor REINDEER(v1.0.2), but also the pseudo-aligners kallisto(0.46.2) and Salmon(v1.5.1), which presumably give an upper bound for the achievable accuracy, since they use an expensive alignment step. For all tools, a *k*-mer size of 19 was used in accordance with the analysis of the simulated data in Seiler *et al.* (2021).

#### 2.5.1 Simulated data

We generated 256 experiments, each with two files containing differentially expressed genes. For this, 75-bp paired-end reads based on 100 randomly picked protein-coding transcripts of the human genome were simulated using the simulator Polyester (Frazee *et al.*, 2015). In these experiments, 10% of transcripts were simulated as differentially expressed, the fold change of the differentially expressed transcripts was 1/4, 1/2, 2 or 4 with equal probability. Four different coverages were used, namely 20, 40, 60 and 80, which means 64 pairs of files for each coverage.

The simulated experiments were evaluated by determining the fold change for the differentially expressed genes using the not normalized expression values of the respective tool. Furthermore, we computed the fold change between the different coverages for the not differentially expressed genes.

#### 2.5.2 Real data

To assess the accuracy with a real dataset, we used data from the Sequencing Quality Control (SEQC) project (SEQC/MAQC-III Consortium, 2014). The SEQC data contains four samples, where Sample A consists of Universal Human Reference RNA (UHRR) and Sample B of Human Brain Reference RNA (HBRR). Samples C and D consist of a mixture of A and B in ratio 3/1 for C and 1/3 for D. The expression of the RNA-seq data was determined by real-time polymerase chain reaction (RT-PCR) for 818 genes. Furthermore, expression values of microarray data for the same samples for 15 984 genes were taken into consideration.

Three evaluation methods were used. First, the Spearman correlation between the ground truth expression values from the RT-PCR and the expression values as determined by the respective tool. Second, the Spearman correlation with the microarray expression values was determined. And third, the mean square error of the expected fold change of the ratio between C and D and fold change calculated from the expression values determined by the different tools was reported. The fold change based on the expression values is based on the ratio of C and D and was calculated by the following formula:

(6) $$\frac{D}{C}=\frac{A_{i}+3B_{i}}{3A_{i}+B_{i}}$$

Where *A_i_* and *B_i_* represent the expression values of a transcript *i* for experiment A and B.

### 2.6 Speed and space consumption

To evaluate the speed and space consumption of Needle, the well-established dataset from Solomon and Kingsford (2016) was used. It consists of 2568 RNA-Seq experiments containing blood, brain, and breast tissue. Similar to our approach in Raptor (Seiler *et al.*, 2021), we excluded all experiments with an average read length below 50 bp. Reads shorter than that are rarely relevant, and this gave us the opportunity to test the minimizer approach with a broader window size. Removing those files left us with 1742 RNA-Seq experiments which have a size of around 6TiB (gzipped FASTQ files).

On this dataset, a preprocessing step was performed before the actual index building, similar to previous analysis (e.g. Marchet *et al.*, 2020; Pandey *et al.*, 2018). This preprocessing step calculates the *k*-mers/minimizers beforehand and removes every *k*-mer/minimizer which does not meet a certain count threshold. The well-established thresholds (Pandey *et al.*, 2018; Solomon and Kingsford, 2016) were used, the thresholds depend on the size of the gzipped file and are either 1 (for ≤300 MB), 3 (for 300−500 MB), 10 (for 500−1000 MB), 20 (for 1−3 GB) or 50 (for >3 GB).

Furthermore, we used a *k*-mer size of 21 as proposed by REINDEER (Marchet *et al.*, 2020).

### 2.7 Using quantification for differential gene expression analysis

One possible application of Needle is to use it as a prefilter to find promising new leads in a research project. As a proof of concept, we analyzed the above-mentioned 1742 experiments by using differential expression to find tissue-specific genes.

We quantify and normalize all known protein-coding genes in these experiments with the (41, 21)-Needle index and consider all genes, which are differentially overexpressed between one tissue compared to the other two tissues according to the t-test using a p-value that is corrected by the number of genes. These differentially expressed genes are then analyzed with ShinyGO (Ge *et al.*, 2019) for a gene ontology analysis.

### 2.8 Extracting experiments based on quantification

Another possible application of Needle is to determine experiments of interest. As a proof of concept and inspired by the analysis of Marchet *et al.* (2020), we searched for overexpression of the breast cancer oncogenes CCND1, ERBB2, FOXM1, MYC in the above mentioned 1742 experiments, which contain 552 breast cancer samples according to the experiment’s metadata.

Overexpression is defined within a sample by considering the mean expression of all protein-coding genes in that sample. If an oncogene has a greater expression than the mean, it is considered overexpressed. The set of samples with an overexpressed oncogenes is then compared to the actual set of breast cancer samples to determine true and false positives, as well as true and false negatives.

## 3 Results

All analyses were performed on a Linux machine (MarIuX64 2.0) with 1TB RAM and an Intel(R) Xeon(R) Gold 6248 CPU (@ 2.50 GHz with 20 cores and 58MB L3 cache). The false positive rate of Needle was set to 0.05 for all analyses, as this is a typically used false positive rate with Bloom filters (Bingmann *et al.*, 2019). In our supplementary, we provided the same results with a rather high false positive rate of 0.3 as suggested by Bingmann *et al.* (2019) in their approach.

### 3.1 Accuracy

For the simulated dataset, Needle was applied with *user-defined thresholds* by using 15 thresholds ranging from 5 to 520. This covers all coverages multiplied with the possible fold changes to see Needle’s performance in the best-case scenario, where the possible expression values are known beforehand. For the SEQC dataset, Needle’s automatic threshold determination was applied, where the number of levels was set to 10 because more levels had little impact on Needle’s performance, as higher levels only store information about minimizers with high counts.

As mentioned before, REINDEER’s query does not return an expression value but all *k*-mer occurrences, hence similar to the definition in Needle we took the median of these occurrences as the expression value. Because REINDEER has an exact mode, REINDEER’s result should be the upper bound of Needle’s accuracy, which only approximates the median.

**Fig. 5.**
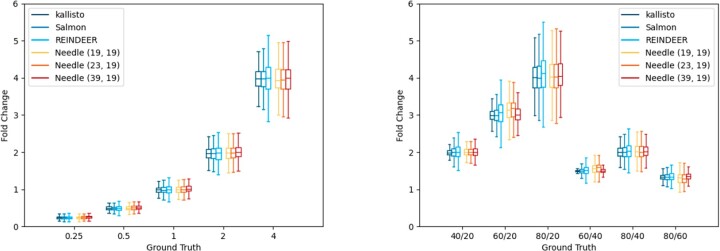
*Left* shows the differential expression comparison and *right* the coverage comparison of Needle, kallisto, Salmon and REINDEER for *k *=* *19 on the simulated dataset. Needle (w,k) represents Needle based on (w,k)-minimizers. The values on the *x*-axis in the left figure represent the ground truth, the expected fold change between differential expressed genes, while the *y*-axis presents the actual measured fold change. The values on the *x*-axis in the right figure represent the ground truth, the actual fold change between coverages, for example 40/20 stands for coverage 40 divided by coverage 20, while the *y*-axis presents the actual observed fold change between coverages

As expected, kallisto and Salmon are the most accurate tools for the simulated dataset, they have the smallest variation and their medians are the closest to the actual fold changes of the differentially expressed genes and the coverages (see Fig. 5). Needle and REINDEER show a similar enough performance. Their medians only differ slightly, and they show a greater variance. Interestingly, Needle seems to perform a bit better than REINDEER as it has a smaller variance in most cases. Furthermore, Needle’s performance with different window sizes is in all instances accurate enough and no big difference between the window sizes can be spotted. Moreover, the variation of all tools increases with the fold change for both the differentially expressed genes and the coverages, indicating that the correct quantification of highly expressed transcripts is more difficult to determine, probably due to repetitive regions.

For the SEQC dataset, we expected kallisto and Salmon to perform better than REINDEER and Needle as they use more information such as the order of the *k*-mers, but surprisingly REINDEER and Needle perform as good as kallisto and Salmon (see Table 1) regarding the Spearman correlation. In some cases, Needle performs even slightly better than the other tools. This is even more surprising as REINDEER uses the same method to determine the expression value of a transcript as Needle, but does so exactly instead of approximately. The better performance of Needle indicates that an approximation might be better equipped to deal with the noise in the data and therefore is more robust.

**Table 1.** Comparison of Needle, kallisto and Salmon for *k *=* *19 on the SEQC dataset

	SEQC	Microarray	MSE
kallisto	80.2	76.3	0.6
Salmon	80.7	76.7	0.6
REINDEER	80.3	76.8	1.3
Needle (19, 19)	80.6	77.1	0.5
Needle (23, 19)	80.5	77.1	0.5
Needle (39, 19)	80.4	76.9	0.5

*Note*: Needle (w,k) represents Needle based on (w,k)-minimizers. SEQC represents the Spearman correlation in percent to the RT-PCR quantification, microarray the Spearman correlation in percent to the miroarray quantification and MSE gives the mean square error of the titration monotonicity transcript-wise.

Additionally, Needle’s normalization is seemingly slightly better than kallisto’s and Salmon’s (see Table 1), but we think this is not a significant difference, especially because the variance for all three tools is around 3. However, it shows that Needle’s normalization works similar to already established normalization methods like TPM. The high mean square error for REINDEER with the SEQC dataset is a result of the missing normalization method. If Needle is performed without a normalization, the mean square error is 1.2 for all window sizes and the variance is much higher. Furthermore, this result underlines the importance of a normalization method, even in cases where the experiments have similar coverages.

As already seen in the simulated dataset, using different window sizes for the minimizers like has such a small impact on the accuracy that the difference is negligible. Hence, the usage of minimizers instead of *k*-mers is completely reasonable.

The similar performance of Needle and REINDEER to kallisto and Salmon shows that using a definition as simple as the median of the minimizer occurrences to determine the expression of a gene is more than reasonable.

### 3.2 Space and speed

While running REINDEER, we encountered multiple problems, which were also reported by other users. While constructing the REINDEER index in its exact mode, we ran into a zlib error when using fewer than 4 threads. But for multiple threads, querying lead to a segmentation fault error. Therefore, we used 1 and 4 thread(s) for the comparison. Moreover, for REINDEER approximation mode using the log option, querying was not possible as it resulted in either an output that did not return all count values, or caused a memory allocation error. Hence, it was not possible to analyze the accuracy of this mode. A run time comparison with all erroneous queries was nonetheless possible, as at least loading the index worked. These loading times are reported for REINDEER’s query, if the query resulted in an error.

Unlike the accuracy analysis on the SEQC dataset, we used 15 levels to avoid giving Needle an advantage by storing too few levels. But even for the biggest Needle index, all IBFs from level 10 onward only had a size of a few MBs. Thus, storing even more levels would have little impact on Needle’s overall performance, which makes this a fair comparison to REINDEER.

Needle outperforms REINDEER in every aspect, it is up to 48 times faster in construction than REINDEER log when using a window size of 41 and in its compressed form 16.4 times smaller (see Table 2). Comparing Needle with a window size of 21 to the exact REINDEER index using 4 threads, Needle is two times faster and its index in compressed form takes less than half of REINDEER’s size. Even compared to REINDEER’s smaller index (REINDEER log), Needle performs better in every version regarding construction time and compressed index size.

Needle (21, 21) has a larger memory footprint than REINDEER because all IBFs are loaded into memory during construction. However, we believe Needle is applicable for most modern use cases as a memory usage of 121 GB can be handled by most modern servers and once the Needle index is created, the memory usage is only a fraction of the built memory usage (see Table 2).

While Needle (21, 21) in its uncompressed form has a larger index size than REINDEER, Needle has the ability to rely on a much smaller index through compression, the usage of a greater window size or the usage of a larger false positive rate (see Supplementary Table S5).

The advantage of a greater window size can be seen in the huge impact it has on the efficiency (see Table 2). An increase of the window size by 4 [(25, 21)-minimizers here, (23, 19)-minimizers in the accuracy datasets], which was shown to be almost as accurate as a window size equaling the *k*-mer size, leads to an improvement in the construction time, the RAM usage and the index size by a factor of 3. For an increase in the window size by 20 [(41, 21)-minimizers here, (39, 19)-minimizers in the accuracy datasets] this speedup factor is at least 12.

Therefore, it is reasonable to use greater window sizes because the small loss of accuracy is exchanged for a huge gain in efficiency.

The advantages of Needle can also be seen in its fast query time (see Table 2). When querying 1000 transcripts, Needle with (w,k)=(41,21) needs 15 s, which is over 100 times faster than REINDEER’s 1, 710 s and still vastly faster than even querying just one transcript with REINDEER, which takes more than 800 s. Even with all *k*-mers (w,k)=(21,21), Needle is over 10 times faster. While we could not run a query with REINDEER log successfully, we were capable of loading the index, which is a necessary step in the search, this took around 9 min. Hence, Needle also outperforms in every version the REINDEER log index.

The number of threads only improves the query performance. Therefore, the performance gain is more visible the more sequences are searched.

Querying Needle with window sizes of 25 and 41 takes around or less than a minute (for *w *=* *25) and less than half of a minute (for *w *=* *41) for all numbers of transcripts. As expected, we can see that the faster loading of a smaller compressed Needle index has less impact with an increasing number of transcripts or the usage of multiple threads, making the uncompressed searches the fastest option for searching 1000 transcripts as the overhead of the compressed version becomes the determining factor.

### 3.3 Identifying tissue-specific genes

In this experiment, we want to examine if the overexpressed genes identified with Needle were correctly annotated to the tissue type of origin. Quantifying and normalizing all protein-coding human transcripts (103, 155 total) took <20 min with one thread (<6 min with four threads).

**Fig. 6.**
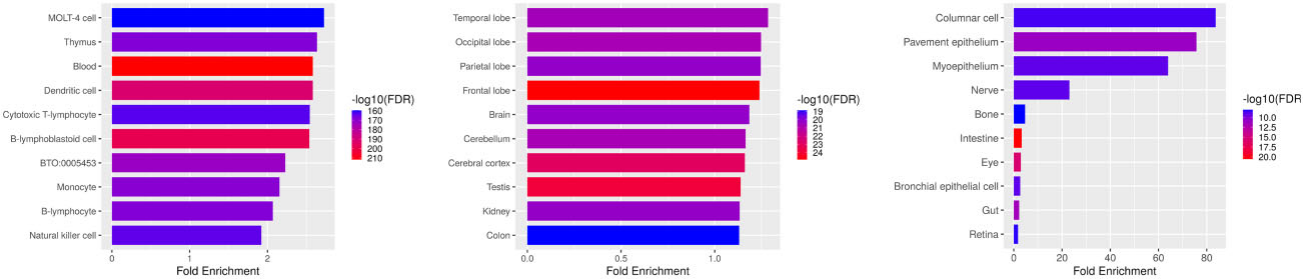
Gene ontology analysis for genes found differentially expressed in blood, brain and breast tissue of 1742 RNA-seq experiments sorted by their fold enrichment. The colors show the false discovery rate in negative log10

The differential expression analysis led to the discovery of 1423 differentially expressed genes for the blood tissue, 2293 for the brain tissue and 90 for the breast tissue. As can be seen in Figure 6, the found genes indeed correspond to known genes related to the tissue of origin. The genes overexpressed in brain tissue samples are associated with the temporal, occipital, parietal and frontal lobe. The overexpressed genes from the blood samples are associated with blood directly but also to tissues involved in leukemia, indicating that the blood tissue samples in the experiments might originate from cancer patients. The overexpressed genes from the breast tissue show a high enrichment for columnar cells, which is reasonable as columnar cell lesions are diagnosed often in breast tissue due to the increase in mammography screening (Logullo and Nimir, 2019).

Moreover, already known associations are found. For example, the association between brain and kidney tissue (Chen *et al.*, 2021) or the association between breast and eye tissue (Houlston and Damato, 1999).

Furthermore, we performed a disease ontology analysis as well and as can be seen in the Supplementary Table S7, the associated genes also correspond to the tissue of origin. This proves that the differentially expressed genes found by Needle are useful for an exploratory analysis.

The point of the performed analysis was not to find new revelations about the blood, brain or breast tissue, but demonstrate a use case of Needle. Unlike kallisto or Salmon, Needle is capable of quantifying thousands of genes in thousands of experiments in minutes. Therefore, if a researcher is interested in finding new associations between different tissues, different diseases or different genomes, Needle can be used as a starting point to find interesting transcripts.

At the same time, this analysis can be seen as a confirmation of already established knowledge. Therefore, Needle can also be used as a sanity check.

### 3.4 Identifying cancer samples within a collection

As shown in Table 3, the false positive rate is low for all oncogenes, but depending on the oncogene the false negative rate is quite high. Nevertheless, the analysis was merely a proof of concept to show that the quantification can be used for identifying a subset of experiments of interest. The good results of the oncogene CCND1 are promising that this is possible. A more in-depth analysis is necessary to determine how oncogenes can more effectively be used to identify breast cancer samples reliably, as it seems apparent that the usage of only one oncogene has limitations.

**Table 3.** Confusion matrix

	CCND1	ERRB2	FOXM1	MYC
True positives	339	267	168	232
False positives	102	58	118	213
True negatives	1088	1132	1072	977
False negatives	213	285	384	320
False positive rate	0.09	0.05	0.1	0.18
False negative rate	0.39	0.52	0.7	0.58

*Note*: The results of finding breast cancer samples by searching for overexpression of the breast cancer oncogens CCND1, ERRB2, FOXM1 and MYC in absolute numbers.

## 4 Discussion

A database is only as good as its utility allows it to be, therefore storing an enormous amount of biological data without the possibility to search through it efficiently makes storing the data almost pointless. Hence, finding a solution to this bottleneck is one of the most important challenges in computational biology today.

In our study, we have shown that it is not necessary to store the exact occurrences of all representative *k*-mers, it is enough to map them to some levels. Thus, all recent research (Bingmann *et al.*, 2019; Harris and Medvedev, 2020; Kitaya and Shibuya, 2021; Lemane *et al.*, 2021; Marchet *et al.*, 2020; Pandey *et al.*, 2018; Seiler *et al.*, 2021; Solomon and Kingsford, 2016, 2018; Sun *et al.*, 2018; Yu *et al.*, 2018) for finding a fast and space efficient data structure to answer in which experiments a transcript is present, can be easily adapted to a tool estimating expression values by storing this data structure multiple times. However, as we demonstrated in our previous study (Seiler *et al.*, 2021), the most efficient data structure at the moment is the IBF.

Furthermore, we confirmed the finding of Zhang *et al.* (2021) that using a simple measure like the median or the interquartile mean [as done by Zhang *et al.* (2021)] of the *k*-mer occurrences results in meaningful expression values. Due to the similarity in robustness to outliers, we would not expect to see a significant difference between using the median or the interquartile mean, but further research would be necessary to confirm this hypothesis.

Moreover, we recommend the usage of minimizers instead of *k*-mers [as we have done already in Seiler *et al.* (2021)]. While there is an accuracy loss by using minimizers, the massive space and speed gain makes this a reasonable approach. Which leads to the question whether using *k*-mers in the contexts of prefilters is still a sensible choice.

We would have liked to also compare Needle to its other competitor Gazelle, but to our knowledge, the software is not publicly available yet. Based on the analysis done by the authors themselves, the Gazelle index size of the 2586 RNA seq files that we used here as the basis for our large dataset is 36.65 GB and is constructed in 9.23 h. It is not apparent to us whether they used the already established cutoffs as well, but assuming they did and assuming that taking $\left\lfloor \frac{1742}{2568}\right\rfloor =0.6$ of their reported size is a lower bound for storing 1742 of the 2586 RNA seq files we used, then we would expect a Gazelle index of size 21.99 GB to be constructed in 5.5 hours. This is longer than the compressed Needle index for *k*-mers and all Needle indices with a bigger window than *k*-mer size. Moreover, the construction of Gazelle takes almost twice as long as even the slowest Needle construction.

Using 0.6 of Gazelle’s size seems a rather generous lower bound: if we compare the REINDEER index sizes reported in this paper to the ones reported in the REINDEER paper (Marchet *et al.*, 2020) for the 2586 RNA seq files, then the index size we determined for the 1742 files make up more than 0.85 of the size for the 2586 files. This is not surprising, as we reduced the dataset by not considering RNA seq files with a smaller read size and therefore a smaller *k*-mer content.

Moreover, another advantage of Needle is that the false positive rate can be set be the user, such that its speed and space consumption can be directly influenced by the level of desired accuracy. Unfortunately, determining the influence of the false positive rate on the actual accuracy is not straightforward because the false positive rate influences the estimation in two ways: finding the levels where the estimation should happen (*x_e_*, *y_e_* in Equation (2)) and determining the number of found minimizers per level (*a_e_*, *b_e_* in Equation (2)).

Even a high false positive rate of 0.3 can be reasonable—see Supplementary Figures S7 and S8 and Table S4, where we found a similar performance on the simulated dataset and a weaker correlation for every Needle version on the SEQC data. Here, we can see for the first time an actual difference in the window sizes, as the accuracy strongly decreases with an increasing window size. This shows that the accuracy depends on the picked (w,k)-minimizers and the false positive rate. (w,k)-minimizers with a greater window size have fewer minimizers for a sequence than (w,k)-minimizers with a smaller window size, therefore one false positive minimizer has a bigger impact for greater window sizes. Further research is necessary to find a formula to estimate the impact of those two factors on the accuracy.

However, even with a greater window size and a high false positive rate, the accuracy results are still good enough for a prefilter and might be a reasonable choice considering the advantages of a higher false positive rate, which is a further decrease in construction and query time, RAM usage and index size. For example, the Needle index (41, 21) with false positive rate 0.3 would need <1 GB of space.

Furthermore, Needle could be further improved by using different false positive rates per level. As the search starts at the highest level and numerous transcripts will be found along the way, increasing the false positive rate of the lower levels should have only a small impact on the accuracy while having a greater influence on the size, as the lower level IBF s are the biggest ones. Before such an improvement is implemented, though, a more profound understanding of the effect of the false positive rate on the accuracy is needed.

Moreover, the memory footprint of Needle could be further improved by loading only a few IBFs to memory at a time, this approach will most probably increases the built time. A more in-depth analysis is necessary to determine the best approach in terms of memory footprint and speed.

Needle is based on unprocessed sequencing files, thereby ignoring any available information that was uploaded alongside the sequencing file, like alignment analysis containing counts for specific transcripts. The advantage of Needle is that it is not limited by limitations of previous analysis, which might miss newly annotated genes, for example. Furthermore, Needle is easily searchable unlike these uploaded analysis files and therefore provides more utility. Nonetheless, it would be interesting to incorporate already existing information into Needle and in this way improving Needle’s accuracy further. Additional research is necessary to find an efficient way to implement such a feature.

Besides improvement possibilities of Needle itself, there is great potential in further researching methods to analyze large collections of sequencing experiments. We showed that within-sample and between-sample analysis are possible and lead to meaningful results, and presented an accurate normalization method for such large collections. But the here presented analysis did not take advantage of the sheer amount of available data. Therefore, further investigation is necessary to determine, if there is an even better method to determine differential gene expression.

## 5 Conclusion and further work

We presented Needle, a fast and space-efficient prefilter for estimating the quantification of very large nucleotide sequences. Through its very fast search, Needle can estimate the quantification of thousands of sequences in a few minutes or even only seconds, paving the way for new analyses that were before either very time and space consuming or not possible at all. For example, one can easily test the differential expression of genes on a huge dataset and reuse previously obtained data by searching through databases like the sequence read archive in a meaningful way. Moreover, such a quick search could also open the door to the usage of machine learning methods working directly with the sequencing data.

We believe that quick searches on the very large sequencing data can open up entirely new analysis methods and therefore are an important step forward to a better understanding of the growing sequencing data.

## Supplementary Material

btac492_Supplementary_Data
