# Supplementary material for: Needle: a fast and space-efficient prefilter for estimating the quantification of very large collections of expression experiments
Source: Bioinformatics. 2022 Jul 8;38(17):4100–8. doi: 10.1093/bioinformatics/btac492 (PMC9438961; doi:10.1093/bioinformatics/btac492)
Supplement: btac492_Supplementary_Data [file btac492_supplementary_data.pdf]

6 Supplementary Information

6.1 Analysis

All analysis can be found here: `https://github.com/MitraDarja/analysis_needle`.

6.1.1 Accuracy

For the simulated data set, we used for Needle the thresholds: 5, 10, 15, 20, 30, 40, 60, 80, 120, 160, 240, 320, 400, 460, 520. For the SEQC data, Needle stored, similar to kallisto and Salmon, only minimizers that can be found in the human transcriptome (specified with the argument –include). No cutoff values were used for Needle or REINDEER in the accuracy analysis. For the exact commands, see above-mentioned repository.

6.1.2 Speed and Space

The data set Needle was preprocessed with ‘needle minimiser‘ and REINDEER with bcalm2, for this data set cutoffs as described above were used.

6.2 Results for a FPR of 0.3

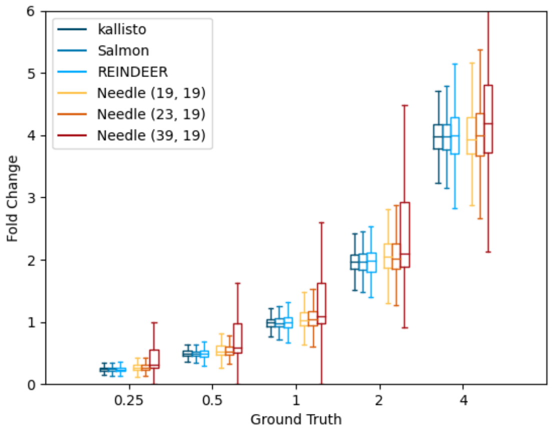

Fig. 7. Differential expression comparison of Needle, kallisto, Salmon and REINDEER for $k = 19$ on the simulated data set. Needle (w, k) represents Needle based on (w, k)-minimizers. The values on the x-axis represent the ground truth, the expected fold change between differential expressed genes, while the y-axis presents the actual measured fold change. Needle was used with a false positive rate of 0.3.

	SEQC	Microarray	MSE
Needle $(19, 19)$	80.2	76.9	0.5
Needle $(23, 19)$	79.5	75.4	0.6
Needle $(39, 19)$	60.1	57.6	1.3

Table 4. Needle results for $k = 19$ and a FPR of 0.3 on the SEQ data set. Needle (w, k) represents Needle based on (w, k)-minimizers. SEQC represents the Spearman correlation in percent to the RT-PCR quantification, microarray the Spearman correlation in percent to the miroarray quantification and MSE gives the mean square error of the titration monotonicity transcript-wise.

6.3 Disease Ontology analysis

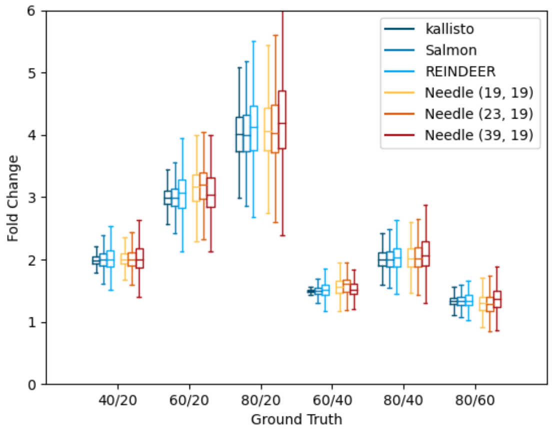

Fig. 8. Coverage comparison of Needle, kallisto, Salmon and REINDEER for $k = 19$ on the simulated data set. Needle (w, k) represents Needle based on (w, k)-minimizers. The values on the x-axis represent the ground truth, the actual fold change between coverages, for example $40/20$ stands for coverage 40 divided by coverage 20, while the y-axis represents the actual observed fold change between coverages. Needle was used with a false positive rate of 0.3.

	Time	RAM	Index Size
Needle $(21, 21)$	100 (108)	17.4 (19.4)	8.9 (11.7)
Needle $(25, 21)$	31 (32)	5.5 (6.2)	2.8 (3.5)
Needle $(41, 21)$	6	1.4 (1.5)	0.7 (0.8)

Table 5. Needle Results for $k = 21$ and a FPR of 0.3 on large real data set. Needle (w, k) represents Needle based on (w, k)-minimizers. Time is in minutes, RAM and Index size in GB. The index size in parentheses is the compressed size.

		Needle $(21, 21)$	Needle $(25, 21)$	Needle $(41, 21)$
1	Time	8 (20)	4 (8)	1 (3)
	RAM	4.4 (9.7)	1.4 (2.9)	0.3 (0.7)
100	Time	16 (33)	5 (12)	2 (3)
	RAM	4.4 (9.7)	1.4 (2.9)	0.3 (0.7)
1000	Time	119 (208)	40 (68)	11 (20)
	RAM	4.4 (9.7)	1.4 (2.9)	0.4 (0.7)

Table 6. Needle Results for $k = 21$ and a FPR of 0.3 on the real large data set for 1/100/1000 sequence(s). Needle (w, k) represents Needle based on (w, k)-minimizers. Results in parentheses are for the compressed Needle version. Time is in seconds and RAM in GB.

	Diseases
Blood	Diamond-Blackfan anemia, Erythropoietic protoporphyria, Relapsing-remitting multiple sclerosis, Frontotemporal dementia, Lupus erythematosus, Cancer
Brain	Carcinoma, Melanoma, Kidney Cancer, Leber hereditary optic neuropathy, Ganglioglioma, Brain disease, Dementia, Toxic encephalopathy, Skin cancer, Liver cancer
Breast	Ductal carcinoma in situ, Bruck syndrome, Pachyonychia congenita, Epidermolysis bullosa, Pemphigus, Pleomorphic adenoma, Osteogenesis imperfecta, Ameloblastoma, Muscular dystrophy, Alopecia

Table 7. Disease ontology for overexpressed genes between different tissues for the $1,742$ sequencing experiments.
